# Income, workload, and any other factors associated with anticipated retention of rural doctors?

**DOI:** 10.1017/S1463423621000839

**Published:** 2022-03-02

**Authors:** Wenjun Yan, Guixiang Sun

**Affiliations:** School of Public Health, Xuzhou Medical University, Xuzhou City, Jiangsu Province, China

**Keywords:** advanced rank, anticipated retention, factors, monthly expenditure, rural doctor, socialization with others

## Abstract

**Objective:**

The turnover of rural doctors, including doctors who leave clinical practice in rural areas, may disrupt the continuity of care. Though strategies had been formulated to address the problems associated with low retention rates, they proved to be unrewarding. This study aimed to investigate how we could anticipate the loss of rural doctors to facilitate their retention in advance.

**Design:**

We conducted a cross-sectional survey and collected data from rural doctors in Jiangsu Province.

**Setting:**

Research on the employment status of target admission graduates in Jiangsu.

**Participants:**

Multi-stage stratified sampling methods were employed to select the respondents in this study. We selected 722 rural physicians, who represented all the rural physicians from Northern, Central, and Southern Jiangsu.

**Measures:**

Factors affecting anticipated rural retention (odds ratios (OR)).

**Results:**

The anticipated rural retention rate was 72.8% for the 722 respondents from Jiangsu province. Economically developed work areas (OR_Central JS_ = 0.501, OR_Southern JS_ = 0.475), a higher monthly income (OR _3000∼_ = 0.584, OR_6000∼_ = 0.255), and an advanced rank among counterparts (OR = 0.507) were protective factors for anticipated rural retention. Risk factors involved the monthly expenditure, mainly for socialization with others (OR = 1.856), working hours of more than 50 hours/week (OR = 2.076), assignment of outpatient work (OR = 1.991), and filing work (OR = 1.544) as the main tasks on a daily basis.

**Conclusion:**

A combination of strategies, including the strengthening of economic incentive as well as the ability to deal with a heavy workload, could increase the recruitment and retention rate in Jiangsu Province.

## Background

An imbalanced geographical distribution is one of the problems affecting the availability of human resources for the provision of health services (WHO, 2019). The improvement of rural and remote primary health care worker recruitment and retention has been highlighted as an issue of vital importance both globally (Cameron *et al.*, 2012; Russell *et al.*, 2013; McGrail *et al.*, 2016) and nationally (Shen *et al.*, 2018). While the recruitment process is critical for the placement of doctors in rural areas, some researchers have argued that there needs to be more focus on factors that impact retention (Hancock *et al.*, 2009). In order to train rural doctors, many countries have set up special rural doctor training projects (Shen *et al.*, 2021), which require a lot of human, financial, and material resources. The low retention rate of rural doctors is considered a wastage of resources. However, the nature of the profession of rural doctors typically necessitates a continuous relationship with residents. A high turnover rate is not conducive to the formation of a stable doctor–patient relationship, which further affects the quality of medical treatment. It has been reported that various combinations of recommendations, such as the establishment of regional medical campuses (Levesque *et al.*, 2018; William *et al.*, 2015) and integration into the local community (Cutchin *et al.*, 1994; Morken *et al.*, 2018), have been implemented in many countries. The setting up of fitness equipment and entertainment facilities (Cameron *et al.*, 2012), education of family physicians in community-based programs (Gruca & Nelson, 2017), and investment in telehealth technology have facilitated the improvement in communication in remote communities (Larsen *et al.*, 2017).

The Chinese government had introduced various recruitment and retention strategies to create and sustain a sufficient rural health workforce. Multi-spot practice (Hu *et al.*, 2012) involved the recruitment of target admission medical students (Ministry of Education and Other Six Departments, 2015) in rural areas. Because most family practitioners remained proximal to the medical education training sites within their province (Fagan *et al.*, 2014), each province recruits its own target admission medical students. According to this policy (Ministry of Education and Other Six Departments, 2015), medical colleges in each province need to recruit rural students from a local province every year. Before entering school, the students should sign contracts with their local bureaus of sanitation. According to the contract, after receiving years of free medical education, graduates should return to their hometowns and serve as rural physicians for at least six years. However, the low rural retention rate, which remains a puzzling problem for the government, inhibits the provision of continuous medical care.

Jiangsu is a developed province; its 2019 Gross Domestic Product (GDP) per capita of 122 398 Ren Min Bi (RMB) is the third highest in China, after Beijing and Shanghai (National Bureau of Statistics of China, 2019), but the maldistribution of doctors continues to remain an issue. There are 590 062 medical health workers in Jiangsu, but only 4.6% have worked in rural areas (National Health and Family Planning Commission, 2019). Of the total population of Jiangsu of 78,063,562, 66.53% has lived in the countryside (Population Census Office of the State Council, 2012). It is important to study the retention of rural doctors to develop policies that would decrease the inaccessibility to Human Resources for Health and ensure the efficient and effective use of resources. One common way to study retention is to ask rural practitioners through surveys whether they expected that they would remain in their current positions (anticipated retention) (Pathman *et al.*, 2013); the results validate the need to estimate anticipated retention in rural health workforce studies. ‘Anticipated retention’ is thus used as a convenient proxy measure for actual retention.

In this study, a province-wide survey was conducted, and information about demographic backgrounds, current career statuses as well as monthly expenditures of doctors recruited to work in rural clinics or hospitals in Jiangsu Province was collected. Their intention regarding the continuation of a rural career was also evaluated as ‘anticipated retention’. The potential factors related to the intention to continue a rural career were explored. On the basis of results, possible solutions to the retention issue were discussed along with the major concerns in policy making with regard to rural health care in China and other countries.

## Methods

A cross-sectional survey was created in April 2018, in accordance with the guidelines of the STROBE (Strengthening the Reporting of Observational Studies in Epidemiology) statement, and the reliable multi-stage stratified sampling method was employed to select the respondents in this study. The following sampling rules were made during the first stage: Jiangsu is an imbalanced province due to the level of economic development in its 13 cities. According to the level of economic development, it can be divided into three parts: Northern Jiangsu (per capita GDP = 75 551 RMB), which includes Xuzhou, Lianyungang, Yancheng, Huaian, and Suqian; Central Jiangsu (per capita GDP = 123 551 RMB), which includes Yangzhou, Nantong, and Taizhou; and Southern Jiangsu (per capita GDP = 167 995 RMB), which includes Suzhou, Wuxi, Changzhou, Zhenjiang, and Nanjing (Provincial Bureau of Statistics of Jiangsu, 2019). Since the retention rate of doctors is related to the level of economic development, two cities were selected randomly in Northern, Central, and Southern Jiangsu; Lianyungang and Yancheng were chosen from Northern Jiangsu, Yangzhou, and Nantong were chosen from Central Jiangsu, while Nanjing and Changzhou were chosen from Southern Jiangsu. During the second stage, two graduates from each chosen city were selected from the list of names (our university recruited rural medical students from all 13 cities in Jiangsu). They were requested to send questionnaires to all the rural physicians through the WeChat working group (this was usually achieved using local rural hospital alliances). This questionnaire was sent to doctors only and not to nurses. To increase the response rate, all the responders were informed that they would receive a red packet when they handed in their questionnaires. Finally, a series of data were collected. Sampling was conducted between 15th August 2018 and 24th August 2019.

The questionnaire sent to the rural physicians included questions regarding demographic information, work conditions, and monthly expenditure items. A key question was asked to all participants to evaluate the anticipated rural retention for these doctors: Do you intend to work at your current workplace until retirement?

Data were collected using the online questionnaire. Cross-table analysis was used for univariate analysis, in which the crude relationship between each factor and anticipated rural retention was evaluated. Binary logistic regression was used to model the relationships between anticipated rural retention and the variables mentioned above. An odds ratio (OR) greater than one was indicative of risk factors and an OR less than one was indicative of protective factors. Statistical analyses were performed using SPSS 23.0 (SPSS Inc., Chicago, IL, USA). Values were considered to be significant if *P* < 0.05.

Ethical approval for this study was obtained from the Ethics Committee at Xuzhou Medical University. (Reference Number: 2018057)

## Results

Of the 827 rural doctors to whom we sent questionnaires, complete responses were obtained from 722 doctors (an effective response rate of 87.3%). Among these respondents, 64.7% (213/329) from Northern Jiangsu reported that they intended to continue a rural career in the future (anticipated rural retention). The anticipated rural retention rates in Central and Southern Jiangsu were 75.5% (142/188) and 80.0% (164/205), respectively. It was hypothesized that the distribution of rural doctors in Jiangsu Province was roughly the same as that of the inhabitants. The weight coefficients (ω Northern Jiangsu = 0.418, ω Central Jiangsu = 0.205, ω Southern Jiangsu = 0.377) of the equation were calculated according to the population distribution of Northern Jiangsu (33 657 400), Central Jiangsu (16 476 700), and Southern Jiangsu (30 372 900) (Provincial Bureau of Statistics of Jiangsu, 2020); the anticipated rural retention rate of Jiangsu Province was 72.8%. The demographic information and work conditions of respondents are shown in Tables 1 and 2.

**Table 1:** Demographic information of 722 rural doctors in Jiangsu (JS)

		*n*	%
Area	Northern JS	329	45.6
	Central JS	188	26.0
	Southern JS	205	28.4
Gender	Male	341	47.2
	Female	381	52.8
Education	Apprenticeship	4	0.6
	Junior school	86	11.9
	Technical secondary school	198	27.4
	Junior college	423	58.6
	Undergraduate	7	1.0
	Master degree and above	4	0.6
Age (year)	<30	264	36.6
	30∼	169	23.4
	40∼	214	29.6
	50∼	63	8.7
	60∼	12	1.7
Professional qualifications	No	63	8.7
	Medical practitioners	410	56.8
	Assistant medical practitioners	23	3.2
	Rural doctor*	98	13.6
	Assistant rural doctor*	102	14.1
	Other	26	3.6
Professional title	Residents	413	57.2
	Attending doctor	192	26.6
	Deputy chief physician	94	13.0
	Chief physician	23	3.2
Monthly income (RMB)	<3000	153	21.2
	3000∼	431	59.7
	6000∼	120	16.6
	9000∼	16	2.2
	12 000∼	2	0.3
Marriage	Unmarried	136	18.8
	Married	576	79.8
	Divorced	1	0.1
	Separated	9	1.2

* The certification of rural doctors and rural assistant doctors was stopped in 2004.

**Table 2:** Work condition of 722 rural doctors in Jiangsu

			*n*	%
Patient number per week	<30		205	28.4
	30∼		168	23.3
	60∼		114	15.8
	90∼		90	12.5
	120∼		145	20.1
Working hours per week (hour)	<40		142	19.7
	40∼		311	43.1
	50∼		269	37.3
Post	Clinician		490	67.9
	Public health doctor		57	7.9
	TCM physician*		42	5.8
	Dentist		10	1.4
	Administrative work		10	1.4
	Other		113	15.7
Main task in daily work (choose three items)	Outpatient service	No	152	21.1
		Yes	570	78.9
	File work	No	472	65.4
		Yes	250	34.6
	Communication	No	403	55.8
		Yes	319	44.2
	Home visit	No	479	66.3
		Yes	243	33.7
	Health education	No	622	86.1
		Yes	100	13.9
Target admission graduates	No		461	63.9
	Yes		261	36.1
Standardized training	No		319	44.2
	Yes		403	55.8
Advanced rank among counterpart	No		626	86.7
	Yes		96	13.3
Higher salary in same rank	No		683	94.6
	Yes		39	5.5

* TCM physician = Traditional Chinese Medicine physician.

**Figure 1:**
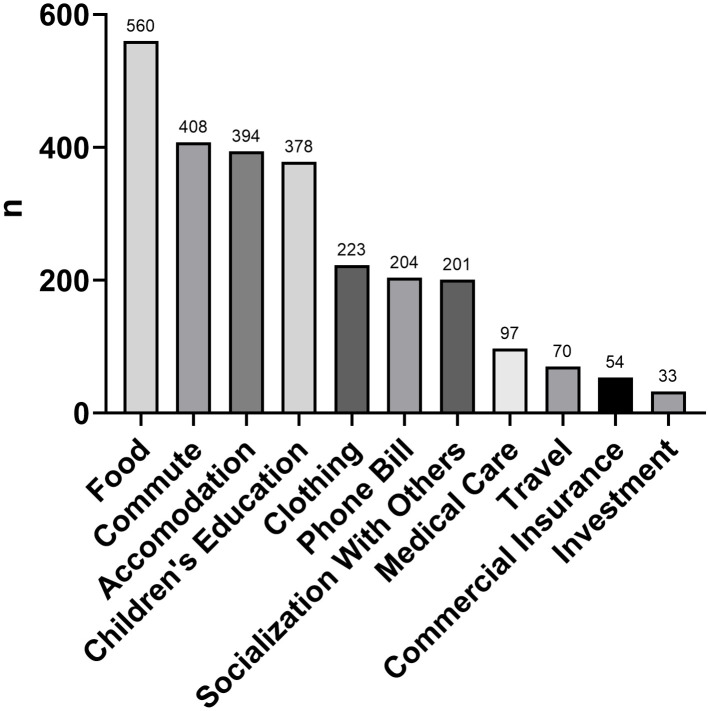
Monthly expenditure items of 722 rural doctors in Jiangsu.

The monthly expenditure of rural doctors was also investigated in this study. Based on Maslow’s hierarchy of needs, human needs arrange themselves in hierarchies of prepotency, that is, the appearance of one need usually depends on the prior satisfaction of another more pre-potent need (Maslow, 1943). The expenditure of rural doctors could reflect their quality of work and life. The respondents were asked to choose the three main monthly expenditure items. We aimed to check whether the main expenditure was spent on low-level needs or high-level needs, as this could potentially reflect the satisfaction and quality of life of rural doctors, and also enable us to determine whether the different expenditures were related to the anticipated retention rate. The top four expenses were food, commute, accommodation, and children’s education (Figure 1).

The characteristics associated with the anticipated retention of doctors in the univariate analysis model were age, education, professional qualifications, professional title, monthly income, marriage, and working hours per week, whether the main tasks assigned daily included outpatient service and filing work, whether target admission graduates were recruited, advanced rank among counterparts, and the expenditure on socialization with others (Table 3).

**Table 3:** Univariate analysis for factors associated with rural doctors anticipated retention

Variables	*χ* ^2^	*P*
Area	16.223	<0.001
Gender	2.289	0.130
Education	12.917	0.024
Age	29.184	<0.001
Professional qualifications	12.367	0.030
Professional title	17.945	<0.001
Monthly income	25.450	<0.001
Marriage	16.957	0.001
Patient number per week	6.420	0.170
Working hours per week	16.665	<0.001
Post	6.650	0.248
Outpatient service	4.753	0.029
File work	12.983	<0.001
Communication with patients	0.783	0.376
Home visit	0.003	0.955
Health education	0.558	0.455
Target admission graduates	7.240	0.007
Standardized training	1.267	0.531
Advanced rank among counterparts	7.182	0.007
Higher salary in same rank	3.306	0.069
Food	0.469	0.493
Commute	0.002	0.962
Accommodation	0.493	0.483
Children’s education	2.963	0.088
Clothing	0.093	0.761
Communication	1.830	0.176
Socialization with others	14.316	<0.001
Medical care	0.004	0.947
Travel	1.061	0.303
Commercial insurance	0.327	0.567
Investment	0.816	0.366

Table 4 shows the relationships between various factors affecting the intention of the respondents to continue a rural career in a binary logistic regression model. In comparison to those in Northern Jiangsu, responders who worked in Central Jiangsu (OR = 0.501, *P* = 0.003) and Southern Jiangsu (OR = 0.475, *P* = 0.002) were more willing to continue to stay. In comparison to those with a monthly income of less than 3000 RMB, the anticipated retention rate of participants with a monthly income of ∼3000 RMB (OR = 0.584, *P* = 0.03) and ∼6000 RMB (OR = 0.255, *P* = 0.001) was higher. Responders who got an advanced rank among counterparts (OR = 0.507, *P* = 0.036) also had a higher anticipated retention rate. In contrast, rural doctors who spent more on socialization with others (OR = 1.856, *P* = 0.002) had a stronger will to leave; those whose working hours per week were greater (OR = 2.076, *P* = 0.007) had a stronger will to leave (compared to less than 40 hours, working hours of more than 50 hours per week can be considered as increased working hours per week); and those who received outpatient work (OR = 1.991, *P* = 0.01) as well as filing work (OR = 1.544, *P* = 0.025) as the main tasks for the day had a negative association with anticipated retention.

**Table 4:** Binary logistic regression model of rural doctors anticipated retention

Variables		*P*	Odds ratio	95% Confidence interval
Area	Central JS	0.003	0.501	0.319, 0.787
	Southern JS	0.002	0.475	0.293, 0.769
Age (year)	30∼	0.103	0.598	0.323, 1.110
	40∼	0.202	0.655	0.341, 1.256
	50∼	0.055	0.427	0.179, 1.020
	60∼	0.999	0	0
Professional qualifications	Medical practitioners	0.340	1.411	0.696, 2.863
	Assistant medical practitioners	0.118	0.167	0.018, 1.574
	Rural doctor	0.402	0.700	0.3041, 1.612
	Assistant rural doctor	0.413	0.728	0.339, 1.559
	Other	0.803	0.860	0.263, 2.812
Professional title	Attending doctor	0.853	0.952	0.565, 1.605
	Deputy chief physician	0.629	0.834	0.400, 1.740
	Chief physician	0.427	0.566	0.139, 2.309
Monthly income (RMB)	3000∼	0.030	0.584	0.359, 0.950
	6000∼	0.001	0.255	0.118, 0.552
	9000∼	0.562	0.641	0.142, 2.886
	12 000∼	0.999	0	0
Marriage	Married	0.233	0.707	0.400, 1.249
	Divorced	1.000	9.827^E8^	0
	Separated	0.731	1.363	0.233, 7.960
Advanced rank among counterparts	Yes	0.036	0.507	0.268, 0.958
Working hours per week (hour)	40∼	0.678	1.120	0.655, 1.914
	50∼	0.007	2.076	1.216, 3.546
Outpatient service	Yes	0.010	1.991	1.177, 3.367
File work	Yes	0.025	1.544	1.057, 2.255
Target admission graduates	Yes	0.289	0.794	0.518, 1.216
Socialization with others	Yes	0.002	1.856	1.253, 2.749

## Discussion

The results of our survey demonstrate that the anticipated rural retention rate was 72.8%; this was similar to the Japanese retention rate of 74% in 2005 (Matsumoto *et al.*, 2005), and was slightly higher than that of 65.97% in Central China (Shen *et al.*, 2018), and was much higher than the retention rate of 57.6% in Thailand in 2018 (Boonluksiri *et al.*, 2018). Although the anticipated retention rate is not as accurate as the actual retention rate, it can still solve some problems. The results of this study showed that the intention to continue to practice as a doctor in the rural areas of Jiangsu was related to the work area; income; monthly expenditure, mainly for socialization with others; advanced rank among counterparts; working hours per week; and assignment of outpatient work and filing work as the main tasks on a daily basis. These results are similar to those in the literature, which indicate that income and hours worked are still among the most important factors influencing recruitment and retention (Witt *et al.*, 2017).

The rural retention rate was significantly higher for physicians receiving higher pay (Elham *et al.*, 2018). Rural doctors with a monthly income of ∼3000 RMB (OR = 0.584) and ∼6000 RMB (OR = 0.255) had a higher anticipated retention rate than those with a monthly income <3000 RMB. However, due to the small sample size (2.5%), we did not identify this trend among responders with a monthly income of ∼9000 RMB and ∼12 000 RMB. According to Maslow’s hierarchy of needs (Maslow, 1943), it is reasonable to hypothesize that if the rural doctors’ incomes are not enough to maintain the needs of a grass-roots life, they would not have the will to improve their quality of service; furthermore, they tended to find a better job. The economic base determines the superstructure, and politics is the concentrated expression of economics. The imbalanced retention rate demonstrated that the improvement in the income of rural medical workers will continue to be the focus of the health system for quite a long time in the future.

Working hours per week is a significant negative contributor to the anticipated rural retention rate. According to our results, doctors who work for more than 50 hours a week are at 2.076 times higher risk of leaving than those who work for less than 40 hours a week. A moderate workload helps to stimulate the best working conditions, but an excessive workload would lead to incompetence, depression, or decreased work performance, increased incidence of errors or accidents, reduced personal satisfaction, and further affect retention rate. In rural areas, besides medical service, filing-related work, such as the setting up of health records for residents, is usually done by rural doctors. Excessive work related to out-patient services and filing can also frustrate rural doctors. An ex-rural doctor complained: ‘I can’t stand it. My job is to set up health records for residents in addition to outpatient services. I have a night shift every two days. I’m so tired. The increased intensity of the workload of rural physicians demonstrates that there is a shortage of rural medical staff due to the low levels of recruitment, which are attributable to the heavy workload in a rural hospital. Something must be done to break the cycle; for instance, increased economic compensation could be provided’ (Witt *et al*., 2017).

As shown in previous studies (Boonluksiri *et al.*, 2018), the working area has been reported to be strongly associated with the retention rate of rural doctors in our study. Rural doctors in Central Jiangsu (OR = 0.501) and Southern Jiangsu (OR = 0.475) were more willing to stay, which might be attributable to the improved infrastructure, provision of higher incomes, and introduction of more welfare schemes in rural hospitals in Central Jiangsu and Southern Jiangsu, compared to those in Northern Jiangsu. An Iranian article revealed that the higher the level of development in a province, the higher the retention rate (Elham *et al.*, 2018), which potentially showed that the rural retention rate is also related to the level of development of the local economy. Doctors and other professionals seek better living standards and professional development. This issue plays a significant role in their retention rate in rural areas (Arah *et al.*, 2008; Chhea *et al.*, 2010).

An interesting finding of our study is that rural doctors with a high socialization-related burden on their monthly expenditure had a stronger will to leave (OR = 1.856); this was seldom reported in previous studies. The Chinese society is a society where interpersonal relationships play an important role. Interpersonal relationships depend on expenditure to some extent. For example, the most common way for Chinese people to connect with friends is to dine with them in a restaurant. Chinese people have the custom of presenting a monetary gift at a wedding of friends or relatives. Spending on these kinds of socialization with others can be a burden for anyone, especially those seeking frequent social connections. On the contrary, integration into the local community was shown to be an important factor affecting rural retention in previous studies (Myroniuk *et al.*, 2016; Morken *et al.*, 2018). The main cause of the difference might be that those whom the doctors spent money on maintaining a social connection with are not locals but friends who worked downtown. This group of rural doctors were well informed about metropolises and were eager to experience life in cities. In the final analyses, they were reluctant to remain in rural areas due to the lack of attraction to rural working conditions. Overall, rural doctors in Jiangsu Province mainly spent money on food, commute, accommodation, and children’s education. Food-related expenditure was still the main expenditure of 77.6% of respondents, which potentially demonstrates that the quality of life of rural doctors in Jiangsu was not high.

According to our results, rural doctors with an advanced rank reported a higher anticipated retention rate (OR = 0.507). As mentioned in a previous study (Morken *et al.*, 2018), the need for a significant other was the most important factor affecting rural retention. A systematic review implied the association between employment structure and rural retention among Australian primary health care workers (Russell *et al.*, 2018). An advanced rank always implies that the doctor has a higher income and higher professional title. In China, the policy of provision of an advanced rank was advertised by some rural hospitals as an attractive condition for recruiting and retaining talents. The results of our study show that this approach is effective.

The recruitment of target admission graduates significantly affects rural retention in univariate analysis, but its significance is lost during binary logistic regression analysis. The relationship between target admission graduates and retention in rural areas was hotly debated in previous studies. Some studies have (Win *et al.*, 2017; Boonluksiri *et al.*, 2018) documented a higher retention rate of medical graduates recruited via the Collaborative Project to Increase Production of Rural Doctors due to their rural background. However, an Australian survey (Gupta *et al.*, 2019) claimed that a rural background was not associated with the timing of introduction to rural work. They highlighted the benefits of rural vocational training. This inconsistency may be due to the different sample sizes in these studies, or because of different national conditions. Target Admission Medical Student Programs are an effective way to solve the problem of recruitment of rural doctors in many countries, as most Target Admission Medical Student Programs require a mandatory service period (Shen *et al.*, 2021). Governments can take several measures during the service period to retain doctors who intend to leave after the expiration of their service period. Another inconsistency may also be due to the sample size of doctors in our study considering marriage. Spousal influence is considered to play a significant role in the recruitment and retention of physicians, as shown in previous studies (Cameron *et al.*, 2012; Giberson *et al.*, 2016; Russell *et al.*, 2018; Levesque *et al.*, 2018). We observed that significant differences in the anticipated retention rate were introduced because of marriage in univariate analysis, but they failed to affect the binary logistic regression model.

The World Health Organization has suggested recommendations to improve the recruitment and retention of health workers in remote and rural areas in four major categories: education, regulation, financial incentives, and professional and personal support for health workers in remote and rural areas (Buchan *et al.*, 2013). The deficiency of economic incentives is still the most important problem in Northern Jiangsu, along with the income of rural doctors and infrastructure. These factors should be taken into consideration by the policymakers next time. Although it is critical to increase the financial incentives, it is only one part of the solution for addressing the health care challenges in rural areas. A concerted effort on the part of national and provincial stakeholders is required to support the rural physician retention strategy. Postgraduate education for rural doctors has been highlighted by many studies (Matsumoto *et al.*, 2005; Gupta *et al.*, 2019). It was treated as a key factor affecting rural retention. We suggest that rural doctors should be trained systematically and periodically, as it could increase the quality of healthcare provided in rural areas.

Recruitment and retention are inseparable from each other. For example, the lack of a workforce is attributable to unfavorable recruitment, and would lead to an excessive workload for existing doctors, and subsequently, to a low retention rate. One possible solution to this issue is a series of comprehensive strategies that would enhance the recruitment rate and retention rate at the same time.

## Limitations

This study had a number of limitations. First, the available data did not include all the variables that could affect retention: some studies had reported that undergraduate exposure (Matsumoto *et al.*, 2005; Boonluksiri *et al.*, 2018) and positive rural learning experiences (Larsen *et al.*, 2017) could effectively increase the recruitment rate. Postgraduate training is a key to increase the rural retention rate (Matsumoto *et al.*, 2005; Gupta *et al.*, 2019). An interview study (Getzin *et al.*, 2016) identified that efforts to enhance health equity and social justice were a major driving force for rural doctors, which brought them to and sustained their work in medicine. Second, we considered the anticipated retention of rural doctors to rural medical care to be a dependent variable. However, though the intention is an alternative indicator of retention, it is not identical to the actual retention of doctors. Follow-up studies are needed to evaluate the actual retention of rural doctors. Third, because of its cross-sectional nature, this study could not determine whether the relationship between each factor and anticipated retention is a causal one.

## Conclusion

Family physicians working with rural populations face complex challenges for achieving quality care goals and sustaining their careers. This study identified different anticipated retention factors that are important to physicians practicing in rural areas. Our findings found that the expenditure on socialization with others as well as an advanced rank among counterparts were related to the anticipated rural retention. In addition, our study enhances the relatively new and growing body of evidence regarding the economic and workload-related factors affecting rural doctors. The important retention patterns highlighted in this survey provide policymakers with direction regarding where to target retention initiatives most effectively, as well as an indication of what they can do to improve rural retention.
